# Factors associated with the goal of treatment in the last week of life in old compared to very old patients: a population-based death certificate survey

**DOI:** 10.1186/1471-2318-14-61

**Published:** 2014-05-07

**Authors:** Tinne Smets, Rebecca Verhofstede, Joachim Cohen, Nele Van Den Noortgate, Luc Deliens

**Affiliations:** 1 Faculty of Medicine and Pharmacy, End-of-life Care Research Group, Vrije Universiteit Brussel & Ghent University, Laarbeeklaan 103, 1090 Brussels, Belgium; 2 Department of Geriatrics, Ghent University Hospital, De Pintelaan 185, 9000 Ghent, Belgium; 3 Department of Public and Occupational Health, EMGO Institute for Health and Care Research, VU University Medical Centre, Amsterdam, The Netherlands

**Keywords:** Palliative care, End-of-life care, Older people

## Abstract

**Background:**

Little is known about the type of care older people of different ages receive at the end of life. The goal of treatment is an important parameter of the quality of end-of-life care. This study aims to provide an evaluation of the main goal of treatment in the last week of life of people aged 86 and older compared with those between 75 and 85 and to examine how treatment goals are associated with age.

**Methods:**

Population- based cross sectional survey in Flanders, Belgium. A stratified random sample of death certificates was drawn of people who died between 1 June and 30 November 2007. The effective study sample included 3,623 deaths (response rate: 58.4%). Non-sudden deaths of patients aged 75 years and older were selected (N = 1681). Main outcome was the main goal of treatment in the last week of life (palliative care or life-prolonging/curative treatment).

**Results:**

In patients older than 75, the main goal of treatment in the last week was in the majority of cases palliative care (77.9%). Patients between 75 and 85 more often received life-prolonging/curative treatment than older patients (26.6% vs. 15.8%). Most patient and health care characteristics are similarly related to the main goal of treatment in both age groups. The patient’s age was independently related to having comfort care as the main goal of treatment. The main goal of treatment was also independently associated with the patient’s sex, cause and place of death and the time already in treatment.

**Conclusion:**

Age is independently related to the main goal of treatment in the last week of life with people over 85 being more likely to receive palliative care and less likely to receive curative/life-prolonging treatment compared with those aged 75–85. This difference could be due to the patient’s wishes but could also be the result of the attitudes of care givers towards the treatment of older people.

## Background

People aged 85 and older form the fastest growing age group in most European countries and their number is predicted to double in the next 20 years [1]. To date, little is known about the type of care that older people of different ages receive at the end of life. Palliative care has been identified as a public health priority worldwide for older people [2,3]. The WHO promotes palliative care as the preferred approach to end-of-life care, irrespective of age. The absence of a palliative or comfort care goal at the time of death in patients suffering from chronic life-limiting diseases is generally associated with poor quality end-of-life care [4].

Palliative care is aimed at improving the quality of life of patients and their families by providing relief from physical, psychological and spiritual problems, while curative treatment is focused on cure or management of a chronic disease and on prolonging life [2-4]. Many chronically ill older people need a mix of both palliative and life-prolonging or curative treatment [5]. However, life-prolonging and curative treatment decreases as the illness progresses and at the end of life the main goal of treatment should be palliative-oriented for most people [4].

Previous studies in Belgium have shown that for approximately 20% of patients a palliative treatment goal is lacking in the last week of life [6]. Recognizing that death is imminent is particularly challenging in the care of older people suffering from slowly progressive or fluctuating long term conditions [5,7]. Studies have shown that the quality of end-of-life care for older people is often suboptimal, especially in hospitals where burdensome interventions aimed at cure or prolonging life are sometimes continued until death [8-10]. Furthermore, a growing body of scientific literature shows that provision of end-of-life care also varies between patients of different ages [11-13].

The objective of this study is to provide a population-based evaluation of the main goals of treatment in the last week of life of people older than 85 compared with those between 75 and 85.

## Methods

This is a secondary analysis of a survey with the primary aim of studying end-of-life practices in Flanders, Belgium. The survey was conducted with the use of data from death certificates in the Flemish speaking part of Belgium. In 2007, we performed a large-scale death certificate study in Flanders, Belgium (approximately 55,000 deaths per year). Questionnaires were sent to the reporting physicians of a representative sample of death certificates received by the Flemish Agency for Care and Health between June 1 and November 30, 2007. We received questionnaires for 3,623 of the 6927 initial cases. From non-response analyses, we found that for 725 cases response was not possible owing to issues of access to the medical file or to patient identification; these cases were removed from the sample. Cases were weighted to be representative of all deaths in Flanders in 2007.

In a first step, we checked whether there we significant differences between the sample and deaths within the general population on the variables sex, age, educational level, marital status, living situation, province of residence, month of death and place of death. Significant differences were found for place of death and cause of death. The sample was subsequently weighted for these variables.

In a second step, the influence of non-response on the representativity of the data was checked. Significant differences were found between deaths where responses had been received and deaths where no response had been received for age, place of residence and cause of death. Cases were subsequently weighted to correct for non-response.

After this double weighting procedure, there were no significant differences between deaths where responses were received and deaths within the general population in 2007.

In the questionnaire, the treating physician was asked whether the death was sudden and unexpected (yes/no). The questionnaire included the question: ‘What was the main goal of treatment in the last week of life?’ with answer categories ‘cure’, ‘life-prolonging’ or ‘comfort’. For this paper, ‘life-prolonging’ and ‘cure’ were concatenated. All non-sudden deaths of persons aged 75 years and older were selected as being in principle eligible for comfort care in the final week of life.

The study protocol has been published elsewhere [14]. Positive recommendations for the anonymity procedure and study protocol were obtained from the Ethical Review Board of the University Hospital of the Vrije Universiteit Brussel, the Ethics Committee of the University Hospital of Ghent University, the Belgian National Disciplinary Board of Physicians and the Belgian Federal Privacy Commission.

### Analyses

Bivariate differences between age groups were tested by Chi-square test. P-values that were less than or equal to 0.05 were considered to indicate statistical significance.

A binary multivariable logistic regression analysis was performed for both age groups to estimate the factors associated with palliative care as the main goal of treatment in the last week of life.

SPSS version 20.0 was used for all statistical computations.

## Results

### Characteristics of non-sudden deaths by age groups

The study sample included 6202 deaths. The response rate was 58.4%. Of all deaths of patients over 75 years old, 1681 were deemed non-sudden. Of those 57.3% were between the ages of 75 and 85 and 42.7% were older than 85 (Table 1). The older group differed in characteristics from the younger group. They were more often female (70.2 vs. 48.9%), widowed (72.1 vs. 38.9%) and of lower education (47.4 vs. 39.5%). Also they more often died from cardiovascular diseases (31.3 vs. 21.3%) and in care homes (54.0 vs. 21.1%).

**Table 1:** Characteristics of non-sudden deaths of patients aged 75–85 compared with patients older than 85*

	**75-85 years**	**>85 years**	**p-value†**
	**N = 964 (57.3)**	**N = 717 (42.7)**	
	**Mean age = 80.2**	**Mean age = 90.7**	
**Sex**			**<0.001**
Female	471 (48.9)	503 (70.2)	
Male	493 (51.1)	214 (29.8)	
**Marital status**			**<0.001**
Widowed	375 (38.9)	517 (72.1)	
Married	473 (49.0)	133 (18.5)	
Single	90 (9.3)	57 (7.9)	
Divorced	27 (2.8)	10 (1.4)	
**Education**			**<0.001**
Primary school	381 (39.5)	340 (47.4)	
High school (not graduated)	182 (18.9)	89 (12.4)	
High school/college	123 (12.7)	73 (10.2)	
Unknown	279 (28.9)	216 (30.1)	
**Cause of death**			**<0.001**
Cardiovascular disease	205 (21.3)	224 (31.3)	
Malignant disease	314 (32.6)	103 (14.4)	
Respiratory disease	136 (14.1)	100 (14.0)	
CVA/stroke	102 (10.6)	62 (8.7)	
Disease of the nervous system	42 (4.4)	29 (4.1)	
Other disease	165 (17.1)	198 (27.7)	
**Place of death**			**<0.001**
Hospital	537 (55.7)	229 (31.9)	
Care home	204 (21.1)	387 (54.0)	
Home	203 (21.1)	85 (11.9)	
Other/unknown/missing	20 (2.1)	16 (2.2)	

*Number of cases (weighted percentages).

† Chi-square test for differences between age groups.

### Patient and health care characteristics by age groups and goal of treatment in the last week of life

Patients between 75 and 85 years more often received life-prolonging or curative treatment than those over 85 years (26.6% vs. 15.8%, p < 0.001). In both age groups, comfort care was more often the main goal of treatment for people in care homes or at home compared with those in hospitals. Other disease characteristics related to receiving comfort care are dying from a malignant disease and being in treatment for a longer period of time (Table 2).

**Table 2:** Patient and health care characteristics by age groups and goal of treatment in the last week of life*

	**75-85 years**			**>85 years**		
	**N = 964 (57.3)**			**N = 717 (42.7)**		
	**Comfort care**	**Life-prolonging or curative treatment**	**p-value**	**Comfort care**	**Life-prolonging or curative treatment**	**p-value**
	**N = 683 (73.4)**	**N = 248 (26.6)**		**N = 574 (84.2)**	**N = 108 (15.8)**	
**Sex**			0.540			**0.005**
Female	337 (72.5)	128 (27.5)		418 (86.7)	64 (13.3)	
Male	346 (74.2)	120 (25.8)		156 (78.0)	44 (22.0)	
**Marital status**			0.505			**0.004**
Widowed	258 (70.9)	106 (29.1)		425 (85.9)	70 (14.1)	
Married	347 (75.6)	112 (24.4)		94 (74.0)	33 (26.0)	
Single	59 (72.8)	22 (27.2)		45 (88.2)	6 (11.8)	
Divorced	19 (73.1)	7 (26.9)		10 (100)	0 (0.0)	
**Education**			0.131			0.141
Primary school	262 (69.7)	114 (30.3)		284 (85.3)	49 (14.7)	
High school (not graduated)	126 (75.4)	41 (24.6)		77 (90.6)	8 (9.4)	
High school/college	86 (72.3)	33 (27.7)		50 (79.4)	13 (20.6)	
Unknown	209 (77.7)	60 (22.3)		163 (81.1)	38 (18.9)	
**Place of death**			**<0.001**			**<0.001**
Hospital	292 (57.1)	219 (42.9)		148 (70.5)	62 (29.5)	
Care home	180 (90.5)	19 (9.5)		338 (89.7)	39 (10.3)	
Home	191 (95.5)	9 (4.5)		74 (91.4)	7 (8.6)	
**Cause of death**			**<0.001**			**<0.001**
Cardiovascular disease	116 (60.4)	76 (39.6)		174 (84.9)	31 (15.1)	
Malignant disease	276 (89.9)	31 (10.1)		93 (93.9)	6 (6.1)	
Respiratory disease	86 (65.6)	45 (34.4)		61 (65.6)	32 (34.4)	
CVA/stroke	65 (65.7)	34 (34.3)		53 (88.3)	7 (11.7)	
Disease of the nervous system	34 (81.0)	8 (19.0)		25 (86.2)	4 (13.8)	
Other disease	106 (66.2)	54 (33.8)		168 (86.2)	27 (13.8)	
**Capacity to make decisions†**			**<0.001**			1.000
Capable	153 (92.7)	12 (7.3)		62 (89.9)	7 (10.1)	
Incapacitated	264 (71.9)	103 (28.1)		269 (89.1)	33 (10.9)	
**Time in treatment for disease that caused death**			**<0.001**			**<0.001**
1-7 days	67 (39.4)	103 (60.6)		90 (65.7)	47 (34.3)	
7 days – 1 month	102 (63.4)	59 (36.6)		103 (80.5)	25 (19.5)	
1 month – 1 year	132 (76.7)	40 (23.3)		98 (90.7)	10 (9.3)	
More than 1 year	69 (94.5)	4 (5.5)		34 (97.1)	1 (2.9)	

*Number of cases (weighted percentages).

† 1242 missing cases.

In the older group, female patients more often had a comfort care goal in the last week of life than did their male counterparts, as had patients without a partner. In the younger group those lacking capacity were more likely to receive life-prolonging or curative treatment at the end of life than were those of that age group with capacity, a difference not found in the older group.

### Factors associated with goal of treatment in the last week of life

After controlling for the confounders sex, cause of death, place of death and time in treatment for the disease, age was independently related to the main goal of treatment in the last week of life. Those in the older group had a 1.61 higher chance (95% confidence interval: 1.20-2.17) of having a comfort care goal in the last week of life as compared with the younger group (not in tables). Other factors associated with comfort care as the main goal of treatment in the last week of life were similar in both age groups (Table 3). The chances of receiving comfort care in the last week of life rather than life-prolonging or curative treatment were in both age groups lower for those dying from non-malignant diseases, for those having been in treatment for the disease for a shorter period of time and for those dying in hospital.

**Table 3:** Differences in age and other factors associated with having a comfort care goal as main goal of treatment*

**Factors related to goal of treatment**	**75-85 years**	**>85 years**
	**OR (95% CI)**	**OR (95% CI)**
**Sex**		
Male	1.00 (1.00-1.00)	1.00 (1.00-1.00)
Female	1.37 (0.90-2.07)	**1.82 (1.03-3.23)**
**Cause of death**		
Cardiovascular disease	**0.25 (0.13-0.48)**	**0.28 (0.09-0.91)**
Malignant disease	1.00 (1.00-1.00)	1.00 (1.00-1.00)
Respiratory disease	**0.28 (0.14-0.55)**	**0.14 (0.04-0.43)**
CVA/stroke	**0.47 (0.23-0.96)**	0.59 (0.16-2.21)
Disease of the nervous system	**0.21 (0.07-0.68)**	0.48 (0.08-2.95)
Other disease	**0.28 (0.14-0.56)**	**0.27 (0.08-0.85)**
**Time in treatment for disease that caused death**		
<1 week	1.00 (1.00-1.00)	1.00 (1.00-1.00)
1 week-1 month	**2.42 (1.48-3.96)**	**2.14 (1.17-3.93)**
1 month-1 year	**2.97 (1.73-5.08)**	**4.23 (1.90-9.43)**
>1 year	**7.43 (2.27-24.35)**	**11.12 (1.07-115.57)**
**Place of death**		
Care home	**8.67 (4.07-18.48)**	**2.91 (1.65-5.15)**
Home	**16.17 (3.13-83.62)**	**7.41 (1.25-44.07)**
Hospital	1.00 (1.00-1.00)	1.00 (1.00-1.00)

*Multivariable logistic regression. Presented figures are odds ratios and 95% confidence intervals.

Significant results are indicated in bold. Independent variables which have no significant relationships are not presented in the table.

## Discussion

This study indicates that for patients aged 75 and above, the main goal of treatment in the last week of life was in a large majority of cases comfort care (77.9%). However, those aged between 75 and 85 were more likely to receive mainly life-prolonging or curative treatment than those older than 85 (26.6% vs. 15.8%) at the expense of comfort care (73.4% vs 84.2%). This age difference persists even after controlling for relevant confounders of sex, cause of death, place of death and time in treatment for the disease. In both age groups, the chances of receiving comfort care in the last week of life were lower for people dying from non-malignant diseases, for those having been in treatment for their disease for a shorter period of time and for those dying in hospital.

Our study used a robust design also pursued in previous studies, including a large representative sample of death certificates and applying a mailing procedure guaranteeing total anonymity for patients and physicians [15,16]. The data collection was completed almost seven years ago. End-of-life care practices may have changed in the meantime.

Although a non-response bias cannot be completely excluded, our non-response survey did not point in that direction. Consequently, we believe our results to be representative for all non-sudden deaths of those older than 75 in 2007 in Flanders, Belgium. As this is a secondary analysis of a survey primarily intended to study end-of-life practices, certain aspects that would have provided a more complete insight, such as the severity of the patient’s condition and their functional status, the content of care in the last week of life, the patient’s wishes for end-of-life care or the existence of an advance care plan were not studied. Additionally, while chronological age is an independent risk factor for adverse outcomes in many conditions, the assessment of frailty in elderly patients may be a superior predictor of outcomes than chronological age in this patient population [17]. Nevertheless, our study is the first to provide robust epidemiological information about the extent to which older people predominantly receive comfort care at the end of life and which factors influence these patients receiving such care.

Finally, the delay between the patient’s death and the study of that death has reached as much as four months in our study as death certificates have to be processed by the proper authorities before they can be made available for research. We therefore cannot exclude the influence of recall bias. However, to address this issue, physicians were encouraged to fill in their questionnaire using the patient files.

Although all deaths in this study were deemed non-sudden and expected by the treating physician, cure or life-prolonging treatment was the main goal of treatment in a substantial number of cases. Controlling for other factors, those above 85 are more likely to have a comfort care goal in the last week of life than are those between 75 and 85. A similar result was found in the Netherlands [18]. There are several possible explanations for this finding: it may suggest a palliative care ethos in the care of those above 85 or, alternatively, it may point to a form of ageism in the sense that age may be used as a criterion for rationing health care [13]. This would imply that the medical system will use more potentially life-saving options, appropriately or not, for those 75–85 than for those older than 85. It may also be that physicians believe that above 85, people are less likely to respond to life-prolonging treatments than are younger old patients or that they feel obliged to ‘do everything’ for younger patients, even though they may find life-prolonging treatments futile for seriously ill patients of any age [12]. It suggests that there is an inclination to pursue life-prolonging treatments as long as the patient is not deemed ‘too old’. Future research should investigate this further.

Irrespective of age group, the chance of receiving comfort care in the last week of life is much lower for older patients who die in a hospital than for those who die in a care home or at home. It is likely that these are older patients sent to hospital precisely for life-saving or curative efforts, for instance in situations where acute care is required. Previous research has indicated that a high number of hospital admissions in older people can, however, be avoided and may be inappropriate [19,20]. A series of complex reasons, including factors relative to the physician, the patient and the family, are usually given for this, the main underlying reason often being the failure to recognize approaching death at the appropriate time and thus to shift treatment towards maintaining comfort [19,20]. Once a patient is referred to a hospital for curative or life-prolonging reasons, the chance to change the focus to palliation may be missed as it can be challenging for hospital staff to distinguish people who can still be treated and recover from their acute situations from those who have reached a point where a shift in focus to palliative or end-of-life care would be more appropriate [21]. Additionally, as reported in previous studies, acute care hospitals often lack a palliative care ethos [22,23]. It is likely that in those cases where comfort is not the main goal of treatment in the last week of life, an opportunity for a transition to palliative care has been missed, even though most older people may be in need of some kind of palliative care [2,3,5]. It would be interesting to study this further in the future.

The likelihood of older people with cancer, compared with those with other chronic diseases, receiving care primarily aimed at comfort is striking. This may be related to the fact that palliative care has historically been focused on cancer patients, who generally have a clearer prognosis than those with non-malignant diseases such as organ failure, stroke or dementia for whom the timing of death often remains unpredictable until it is very close or who may die unexpectedly before palliative care can be started [5,7,24,25].

## Conclusions

Although improving the accessibility of palliative care for older people has been identified as an international public health priority, our findings show that even in the last week of life comfort care is not the main goal of care for a substantial proportion of older people, even among those over 85 [2,3]. These findings warrant more attention to the palliative care needs of older patients, perhaps particularly those between 75 and 85 who seem to be at a higher risk of receiving burdensome curative or life-prolonging interventions, possibly at the cost of their comfort, than those over 85. The principles of comfort therapy should be integrated into the daily decision making, especially in hospital. Further research is needed to better understand the needs of patients of different ages at the end of life and how age influences end-of-life care.

## Competing interests

The authors declare that they have no competing interests

## Authors’ contributions

The data presented in this article are based on a nationwide post-mortem survey using death certificates. LD was project supervisor. TS analysed the data and wrote the manuscript. All authors contributed to data analysis and commented critically on several drafts of the manuscript, including the final version. All authors had full access to all of the data (including statistical reports and tables) in the study and can take responsibility for the integrity of the data and the accuracy of the data analysis. All authors read and approved the final manuscript.

## Pre-publication history

The pre-publication history for this paper can be accessed here:

http://www.biomedcentral.com/1471-2318/14/61/prepub
